# Cellular Expression Profile for Interstitial Cells of Cajal in Bladder - A Cell Often Misidentified as Myocyte or Myofibroblast

**DOI:** 10.1371/journal.pone.0048897

**Published:** 2012-11-07

**Authors:** Weiqun Yu, Mark L. Zeidel, Warren G. Hill

**Affiliations:** 1 Laboratory of Voiding Dysfunction, Department of Medicine, Beth Israel Deaconess Medical Center, and Harvard Medical School, Boston, Massachusetts, United States of America; 2 Division of Nephrology, Department of Medicine, Beth Israel Deaconess Medical Center, and Harvard Medical School, Boston, Massachusetts, United States of America; 3 Division of Matrix Biology; Department of Medicine, Beth Israel Deaconess Medical Center, and Harvard Medical School, Boston, Massachusetts, United States of America; Centro Cardiologico Monzino, Italy

## Abstract

**Background:**

Interstitial cells of Cajal (ICC) have been identified in urinary bladder of several species, but their presence in mice remains uncertain. Meanwhile, dozens of reports indicate that dysregulation of connexin 43 plays an important role in bladder overactivity, but its localization has not been clearly defined, with reports of expression in either the smooth muscle or in myofibroblasts. We recently identified a population of ectonucleoside triphosphate diphosphohydrolase 2 (NTPDase2) positive cells that resemble ICC and are distinct from smooth muscle, fibroblasts, myofibroblasts and neurons. Thus we sought to define more clearly the molecular signature of ICC and in doing so resolve some of these uncertainties.

**Principle findings:**

Immunofluorescent localization revealed that NTPDase2-positive cells lie closely adjacent to smooth muscle but are separate from them. NTPDase2 positive cells exhibited co-localization with the widely accepted ICC marker - c-kit. They were further shown to co-localize with other ICC markers CD34 and Ano1, but not with mast cell marker tryptase. Significantly, they show convincing co-localization with connexin 43, which was not present in smooth muscle. The identity of these cells as ICC was further confirmed by the presence of three mesenchymal markers – vimentin, desmin, and PDGFβ receptor, which indicates their mesenchymal origin. Finally, we observed for the first time, the presence of merlin/neurofibromin 2 in ICC. Normally considered a neuronal protein, the presence of merlin suggests ICC in bladder may have a role in neurotransmission.

**Conclusions:**

NTPDase2 positive cells in mice bladder are ICC, which can be defined by the presence of c-Kit, CD34, Ano1, NTPDase2, connexin 43, vimentin, desmin, PDGFβ receptor and merlin/NF2. These data establish a definitive molecular expression profile, which can be used to assist in explorations of their functional roles, and the presence of NTPDase2 suggests that purinergic signaling plays a role in regulation of ICC function.

## Introduction

In the gastrointestinal tract, interstitial cells of Cajal (ICC) function as pacemakers, neurotransmitter transducers, and mechanosensors that respond to physical and chemical signals, and thereby modulate smooth muscle contractility [1], [2], [3], [4]. Alterations in ICC function have been linked to more than a dozen gastrointestinal diseases [5], [6]. In the last decade, novel ICCs have also been identified in the urinary bladder in several species, including guinea pigs, rats, and humans [7], [8], [9], [10], [11], [12]. Unlike ICC in gut, the function of ICC in bladder is poorly understood, but emerging data indicates that they too, are implicated in several bladder diseases. These disorders offer the chance to gain insights into ICC functioning. In megacystis-microcolon intestinal hypoperistalsis syndrome (MMIHS), a congenital lethal disease in newborns, patients are unable to void spontaneously and have a massively dilated bladder. It is thought that the lack of ICC in the MMIHS bladder is responsible for this lethal voiding dysfunction [8].

Proto-oncogene c-Kit (C-kit, tyrosine-protein kinase Kit, or CD117) is a receptor tyrosine kinase (RTK) expressed on the surface of hematopoietic as well as other cell types such as mast cells. Signaling through c-kit plays a role in cell survival, proliferation, and differentiation, and gain of function mutations in this protein are associated with multiple tumors [13], [14], [15], [16]. In the digestive tract, c-kit is used as the “gold standard” for identification of ICC. C-kit has also been identified in urinary bladder in guinea pig, rat and human, and further functional characterization has suggested these c-kit-positive cells are like the ICC in gastrointestinal tract [11], [17], [18], [19], [20], [21]. In the mouse urinary tract, there has been some confusion about the presence of c-kit in bladder ICC. Pezzone and co-authors reported c-kit positive cells in ureter, but not in bladder [22]. Meanwhile, McCloskey *et al.* identified c-kit positive cells in both wild-type and *W/Wv* (c-kit mutant) mice [23], while other investigators have failed to find c-kit in mouse bladder at all [24], [25].

ICC are stellate-like cells with long dendrites or spikes. They have close contacts with nerve varicosities and smooth muscle cells and form gap junctions with each other, which provide a route for the diffusion of low molecular weight materials as an important intercellular signal communication pathway between these types of cells. Thus gap junctions have a crucial role in mediating the synchronized contraction of smooth muscle cells. Nemeth *et al.* reported that gap junction protein connexin 43 is present in ICC with convincing co-localization of c-kit. ICC could be seen to form a three-dimensional network in the normal colonic bowel wall and lack of connexin43 expression in the aganglionic bowel of Hirschsprung's disease (HD) may be partly responsible for the smooth muscle motility dysfunction in HD patients [26]. It has remained unclear as to whether ICC in the urinary bladder also express connexin 43, or where connexin 43 is located. While dozens of studies indicate that connexin 43 plays a crucial role in modulating bladder overactivity, its precise localization is still the subject of debate and attributed mostly to bladder smooth muscle cells and/or myofibroblasts. We therefore sought to define more clearly the molecular signature of ICC and in doing so resolve some of these uncertainties [27], [28], [29], [30], [31], [32].

We recently identified a population of cells in mouse bladder that express ectonucleoside triphosphate diphosphohydrolase 2 (NTPDase2), an ectonucleotidase that degrades ATP/UTP to ADP/UDP and further to AMP/UMP [33]. These NTPDase2-positive cells are a unique subset of cells in bladder that exhibit narrow elongated and branched cell processes and clearly wrap around smooth muscle cell bundles. They are also expressed in a sub-region of the lamina propria that is immediately adjacent to the bladder smooth muscle layer and seems to be a natural extension from between the smooth muscle spaces. Intriguingly our data indicated they were distinct from smooth muscle, fibroblasts, myofibroblasts and neurons, indicating they might be ICC [33].

In this study we have used confocal immunofluorescent microscopy of multiple cellular markers to define the identity of these cells. Our data demonstrate that they are ICC by the usual accepted definitions and that ICC in mouse bladder can be clearly defined by the presence of c-Kit, NTPDase2, CD34, Ano1, connexin 43, vimentin, desmin, PDGF receptor and merlin/NF2. These data establish a molecular expression profile for ICC in mouse bladder, which can be used to assist in explorations of their functional roles in future.

## Materials and Methods

### Materials

Unless otherwise specified, all chemicals were obtained from Sigma (St. Louis, MO) and were of reagent grade or better.

### Animals

Mice used in this study were C57BL/6J mice (19–21 g) from Charles River Laboratories (Wilmington, MA). Mice were euthanized by inhalation of 100% CO2. After euthanasia and thoracotomy, the bladders were rapidly excised and processed as described below. All animal studies were carried out with the approval of the Beth Israel Deaconess Medical Center Institutional Animal Care and Use Committee (Protocol **#**051-2009).

### Antibodies and labeled probes

Affinity-purified polyclonal sheep anti-NTPDase2 antibody (AF5797) was purchased from R&D systems (Minneapolis, MN). Affinity-purified monoclonal rat anti-CD34 [MEC14.7] antibody (ab8158) and monoclonal rabbit anti-TMEM16A [SP31] antibody (ab64085) were purchased from Abcam (Cambridge, MA). Affinity-purified polyclonal rabbit anti-c-kit antibody (A0501), affinity-purified polyclonal rabbit anti-connexin 43 antibody (C0158), affinity-purified polyclonal rabbit anti-vimentin antibody (C0390), affinity-purified polyclonal rabbit anti-desmin antibody (C0171), affinity-purified polyclonal rabbit anti-PDGFβ antibody (B7194), affinity-purified polyclonal rabbit anti-merlin antibody (A8046) were purchased from Assay Biotechnology (Sunnyvale, CA). Affinity-purified polyclonal goat anti-mast cell tryptase (G-12) antibody (sc-32474) was purchased from Santa Cruz Biotechnology (Santa Cruz, CA). Affinity-purified monoclonal mouse anti-αSMA antibody is a kind gift from the laboratory of Dr. Raghu Kalluri (Beth Israel Deaconess Medical Center). Secondary donkey anti-rabbit/sheep/goat/mouse antibodies conjugated to Alexa 488/546, and Topro-3 were purchased from Invitrogen-Molecular Probes (Carlsbad, CA).

### Immunofluorescence analysis

Excised bladders were fixed in 4% (w/v) paraformaldehyde dissolved in 100 mM sodium cacodylate (pH 7.4) buffer for 2 h at room temperature. Alternatively, tissue was fixed in 4°C methanol for 10 min. Fixed tissue was cut into small pieces with a razor blade, cryoprotected with 30% sucrose solution (w/v), frozen, sectioned (5 µm), and incubated with primary antibodies (1∶50–1∶500 dilution) overnight at 4°C as described previously [33]. After washing away unbound primary antibody, the sections were incubated with a mixture of Alexa 488-conjugated secondary antibody (diluted 1∶100), Alexa 546-conjugated secondary antibody (diluted 1∶100), and Topro-3 (1∶1,000). The sections were washed with PBS, postfixed with 4% (w/v) paraformaldehyde, and mounted under coverslips with p-diaminobenzidine-containing mounting medium. All immunofluorescent localization data shown are representative images of staining performed on at least three individual bladders.

### Scanning laser confocal analysis

Imaging was performed on a Zeiss LSM-510 confocal microscope equipped with argon and green and red helium-neon lasers (Thornwood, NY). Images were acquired by sequential scanning with a 63× (1.4 numerical aperture) planapochromat oil objective and the appropriate filter combinations. Serial sections were captured with a 0.25 µm step size. The images (1024 & 1024 pixels) were saved as TIFF files. Serial sections were projected into one image using LSM-510 software. The contrast level of the final images was adjusted in Adobe Photoshop, and the images were imported into Adobe Illustrator CS3.

## Results

**Figure 1:**
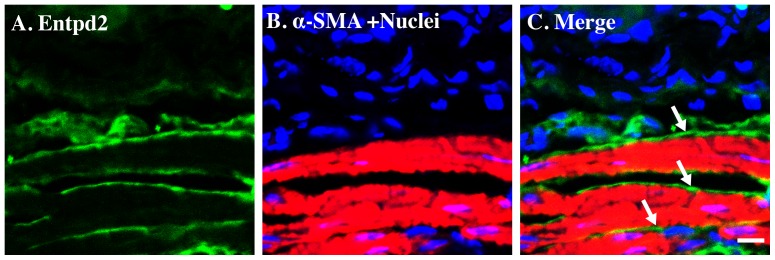
NTPDase2 immunostaining does not co-localize with α-SMA in mice bladder smooth muscle. Cryosections of mouse bladders were labeled with antibodies to NTPDase2 (A. green), α-SMA (B. red) and Topro-3 to label nuclei (B. blue). Color merged panels are shown on the right (C). White arrows indicate distinct NTPDase2 staining next to bladder smooth muscle cells. White scale bars = 10 µm.

Immunostaining of frozen bladder sections was performed for multiple proteins of interest. Figure 1 shows the spatial relationship between ectonucleotidase NTPDase2 and bladder smooth muscle stained with antibody to α-smooth muscle actin (αSMA, red). NTPDase2-positive cells (green) occur in close physical proximity to smooth muscle cells with clear evidence of elongated cell processes. NTPDase2 is also expressed in the stromal compartment next to the detrusor [33]. Arrows in the merged image show that despite lying adjacent to smooth muscle cells, the green signal remains spatially separate from αSMA.

**Figure 2:**
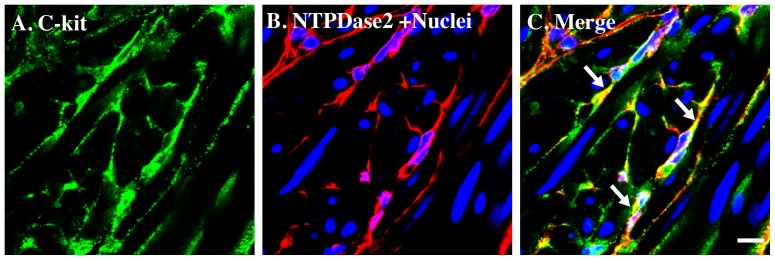
NTPDase2 immunostaining co-localize with c-kit in mice bladder. Cryosections of mouse bladders were labeled with antibodies to NTPDase2 (B. red), c-kit (A. green) and Topro-3 to label nuclei (B. blue). Color merged panels are shown on the right (C). Merged signals of NTPDase2 and c-kit are shown as yellow (C). White arrows indicate representative NTPDase2/c-kit co-localization. White scale bars = 10 µm.

Confocal immunofluorescent laser scanning microscopy revealed that spindly NTPDase2-positive cells were dispersed between smooth muscle cell bundles and densely occupied the lamina propria next to the muscle layer [33], indicating morphological characteristics resembling those of ICC. To confirm this, we stained the same cells with a c-kit antibody, and both signals showed excellent co-localization (Fig. 2). We only observed c-kit staining in NTPDase2-positive cells, and not in smooth muscle or urothelial cells, indicating that the NTPDase2-positive cells in mouse bladder are likely to be ICC.

**Figure 3:**
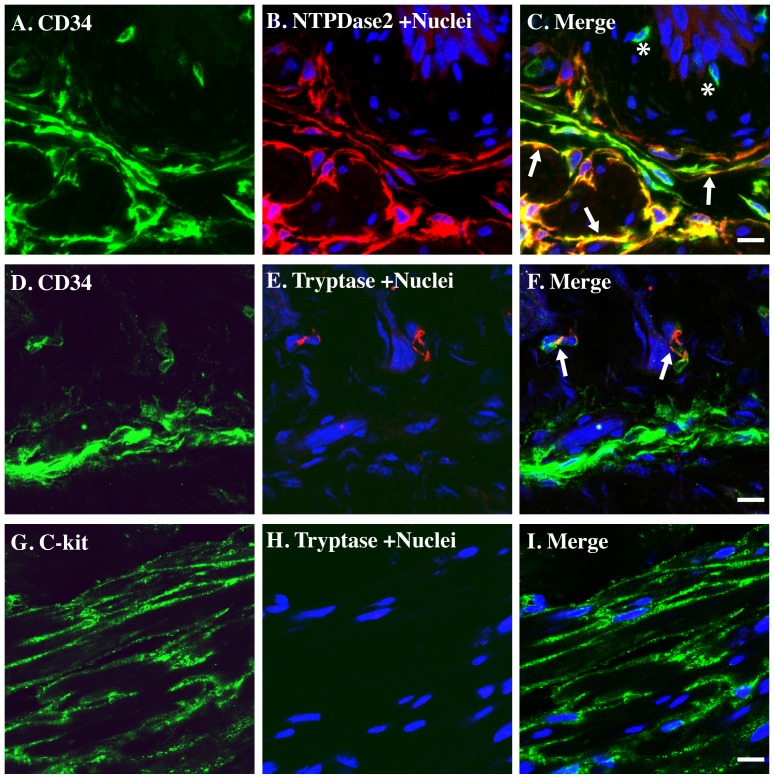
NTPDase2 co-localizes with CD34 but not tryptase in mouse bladder. Cryosections of mouse bladders were labeled with antibodies to CD34 (A, D. green), NTPDase2 (B. red), tryptase (E. red), c-kit (G. green), and Topro-3 to label nuclei (B. E. H. blue). Color merged panels are shown on the right (C, F, I). Merged signals are shown as yellow (C. F. I). White arrows indicate representative NTPDase2/CD34 co-localization (C) or CD34/tryptase co-localization (F); White asterisks indicate CD34 positive cells with no NTPDase2 staining (C). White scale bars = 10 µm.

To extend this initial characterization we stained tissue sections with CD34. CD34 is an important adhesion molecule. It has been identified in hematopoietic cells, endothelial cells, mast cells, as well as being reported in ICC [34], [35], [36], [37]. CD34 antibodies strongly stained the NTPDase2-positive cells (Fig. 3 A–C). CD34 also stained a few NTPDase2-negative cells in lamina propria beneath the urothelium, possibly indicating hematopoietic cells or mast cells. It has been shown that both c-kit and CD34 also stain mast cells in addition to ICC. To exclude the possibility that NTPDase2-positive cells are mast cells, we stained bladder tissue sections with tryptase. The tryptase signal co-localized with the CD34 signal beneath the urothelium in lamina propria, but not in all cells (Fig. 3 D–F). Importantly, c-kit positive cells wrapping around muscle bundles are typtase negative (Fig. 3 G–I). These data strongly suggest that NTPDase2-positive, c-kit and CD34 positive but tryptase negative cells are ICC in mouse urinary bladder tissue.

**Figure 4:**
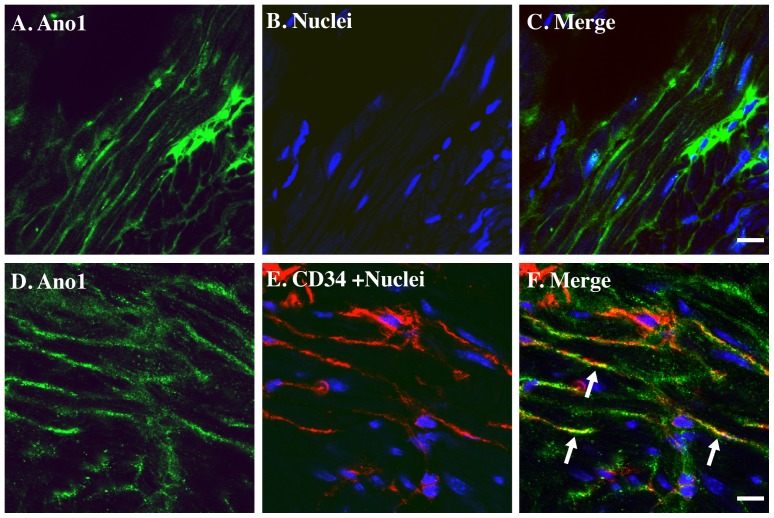
Ano1 co-localizes with CD34 in mouse bladder. Cryosections of mouse bladders (methanol fixation) were labeled with antibodies to Ano1 (A. D. green), Cd34 (E. red) and Topro-3 to label nuclei (B. E. blue). Color merged panels are shown on the right (C. F). Merged signals of Ano1 and CD34 are shown as yellow (F). White arrows indicate representative Ano1/CD34 co-localization. White scale bars = 10 µm.

Recently, anoctamin 1 (Ano1, DOG1, TMEM16A) has been recognized as a selective marker of ICC [38]. Ano1 is a Ca^2+^ - activated Cl^−^ channel and its expression in ICC is fundamental for slow wave activity in gastrointestinal muscles [39]. Interestingly, we observed Ano1 signal in mouse bladder tissue between muscle bundles with a fibrous structure (Fig. 4 A–C), and this signal has nice co-localization with CD34 signal (Fig. 4 D–F). These data provide additional confirmatory evidence that these cells are urinary tract ICC.

**Figure 5:**
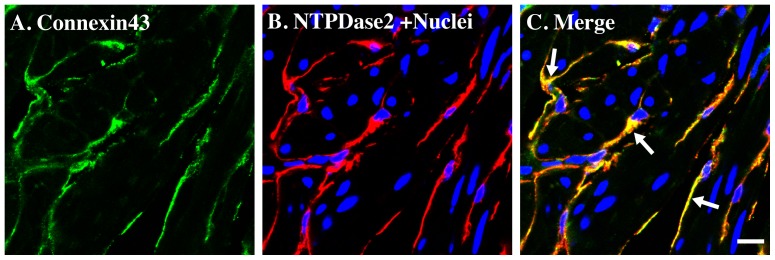
NTPDase2 immunostaining co-localize with connexin 43 in mice bladder. Cryosections of mouse bladders were labeled with antibodies to connexin 43 (A. green), NTPDase2 (B. red) and Topro-3 to label nuclei (B. blue). Color merged panels are shown on the right (C). Merged signals of NTPDase2 and connexin 43 are shown as yellow (C). White arrows indicate representative NTPDase2/connexin 43 co-localization. White scale bars = 10 µm.

NTPDase2-positive cells also exhibited convincing colocalization with connexin 43 (Fig. 5) and interestingly there was no evidence for broader expression of connexin 43 in smooth muscle itself, or any other types of cells in bladder. This data supports the finding of c-kit and connexin 43 co-localization in colonic bowel ICC [26], indicating a number of similarities between bladder and gastrointestinal tract ICC.

**Figure 6:**
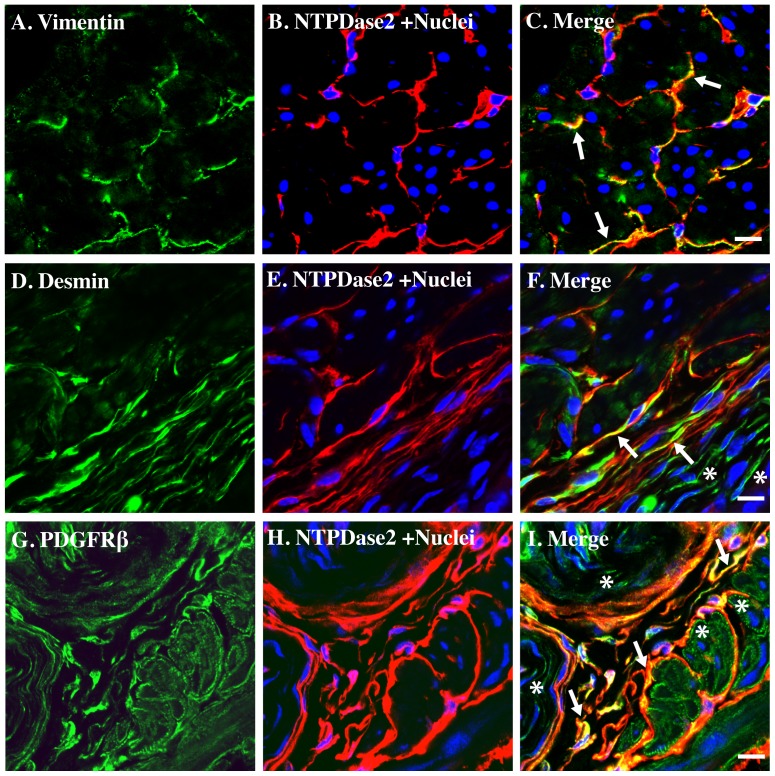
NTPDase2 immunostaining co-localize with vimentin, desmin, and PDGFβ receptor in mice bladder. Cryosections of mouse bladders were labeled with antibodies to vimentin (A. green), desmin (D. green), PDGFβ receptor (G. green), and NTPDase2 (B. E. H. red) and Topro-3 to label nuclei (B. blue). Color merged panels are shown on the right (C. F. I). Merged signals of NTPDase2 and vimentin, desmin, and PDGFβ receptor are shown as yellow (C. F. I). White arrows indicate representative co-localization. White asterisks indicate non-co-localized signal of smooth muscle (I) and fibroblasts (F. I). White scale bars = 10 µm.

Vimentin is the major cytoskeletal component of mesenchymal cells and is often used as a marker of mesenchymally derived cells. Because of this it is also often used as an ICC marker and has been reported in guinea pig bladder ICC and ICC in other organs [12], [40], [41], [42]. Immunostaining showed co-localization of NTPDase2-positive cells with vimentin (Fig. 6A–C). Vimentin antibody also labels NTPDase2-negative cells in the lamina propria (not shown in the photo) and these are likely to be myofibroblasts and/or fibroblasts. Desmin is another cytoskeletal marker closely related to muscle tissue development and differentiation. NTPDase2-positive cells also showed partial co-localization with desmin signal, and like vimentin, labeled other cells in the lamina propria that might be myofibroblasts (Fig. 6 D–F, lower right quadrant). PDGF is a potent mitogen for cells of mesenchymal origin. PDGF receptor plays important roles in driving mesenchymal proliferation, migration, differentiation, and in disease states such as fibrosis. Recently PDGF receptors (PDGF-R) have been reported in ICC in gastrointestinal tract [43], [44]. Consistent with these reports, we detected strong PDGFβ-R signals in NTPDase2-positive cells. PDGFβ-R was also expressed in other cell types, like smooth muscle cells and possibly fibroblasts but generally staining was much weaker (Fig. 6 G–I).

**Figure 7:**
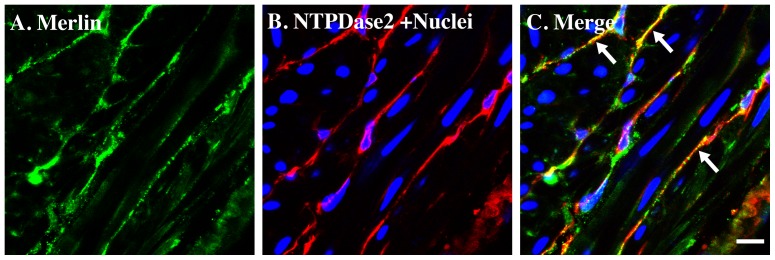
NTPDase2 immunostaining co-localize with merlin/NF2 in mice bladder. Cryosections of mouse bladders were labeled with antibodies to merlin/NF2 (A. green), NTPDase2 (B. red) and Topro-3 to label nuclei (B. blue). Color merged panels are shown on the right (C). Merged signals of NTPDase2 and merlin/NF2 are shown as yellow (C). White arrows indicate representative NTPDase2/merlin co-localization. White scale bars = 10 µm.

Gastrointestinal stromal tumor (GIST) is one of the most common mesenchymal tumors of the gastrointestinal tract. Tumorigenesis is usually associated with gain of function mutations in the c-kit gene and activation of inappropriate tyrosine kinase signaling (∼95%). Activating mutations in other receptors can also result in GIST e.g PDGF-Rα. In recent years, investigators have studied the relationship between GIST and merlin (also known as neurofibromin 2, NF2). Merlin is a membrane-cytoskeletal marker that is mainly located in adherens junctions [45], [46], [47]. It is predominantly found in nerve tissue and functions as a tumor suppressor. Several recent studies suggest that merlin/NF2 could be located in c-kit positive GIST cells [48], [49], [50]. Therefore we were interested to see whether we could detect its expression in bladder ICC. Our data indicate that this protein is expressed in NTPDase2-positive cells in mouse bladder with convincing co-localization of these two proteins (Fig. 7).

## Discussion

ICC were originally discovered as a morphologically distinct cell in gastrointestinal tract [51], [52]. They now have a widely accepted role as a ‘pacemaker’ cell responsible for generating slow waves involved in peristalsis. ICC or ICC-like cells are now being described in many other muscular organs [7], [41], [53], [54], [55], but their functions in these systems are much less clear. The urinary bladder has been reported to possess ICC in several species, but there is some question as to whether they are universally expressed in mammals or have similar functions to their counterparts in the GI tract [22], [23], [24], [56].

In this study, we have clearly demonstrated the presence of ICC marker c-kit, in NTPDase2-positive cells of mouse bladder. NTPDase2 is not expressed by fibroblasts, myofibroblasts, neurons or smooth muscle [33], therefore colocalization with c-Kit identifies it as another specific marker for ICC in bladder. The localization of NTPDase2 expression in ICC cells raises an interesting question as to whether purinergic signaling plays a role in the regulation of their function. Multiple purinergic receptors have been reported in ICC, including P2X_2/5_, P2Y_4_ and P2Y_1_ [57], [58], [59]. In a c-kit mutant mouse (W/Wv), purinergic contraction of bladder smooth muscle has been significantly up-regulated, indicating an intriguing relationship between c-kit signaling pathway and purinergic signaling pathways [23]. The presence of purinergic receptors in ICC and the clear functional importance of purines in bladder pathophysiology, suggests that NTPDase2/purinergic signaling might be important in regulating ICC pacemaker activity or mechanosensory function. Mechanical stretch of cells causes release of ATP [60], and mechanical stretch of ICC regulates pacemaker activity and smooth muscle contraction in both stomach and bladder [1], [21]. Furthermore, the presence of connexin 43 in ICC provides an ideal pathway for ATP and other purines to communicate through connexin hemichannels or intercellular gap junctions [61], [62]. Our findings suggest that NTPDase 2 might play an important regulatory role in ICC purinergic signaling, possibly by temporally and spatially limiting P2-receptor exposure to nucleotides and nucleosides.

There has been some controversy as to the existence of c-kit positive cells in mouse bladder and our own experience has been that many commercially available c-kit antibodies fail to immunostain frozen sections [33]. This is possibly due to variants or isoforms of c-Kit expressed in different tissues and/or substantial species heterogeneity (http://www.uniprot.org/uniprot/P05532). In this study, we co-localized seven putative ICC related markers with NTPDase2-positive cells in mice bladder (Table 1). In addition to c-kit we show these cells also express strong CD34, a protein that also often functions as a mast/stem cell marker, which is consistent with c-kit as a mast/stem cell growth factor receptor, indicating a possible functional relationship.

**Table 1:** Summary of expression profile of molecular markers in bladder ICC.

Markers	Bladder ICC	Myofibroblast/fibroblast	Smooth muscle	Neuron	Mast cell
NTPDase1 (33)	−	−	+	−	
NTPDase2(33)	+	−	−	−	−
Nt5e (33)	−	−	+	−	
FSP1 (33)	−	+	−	−	−
αSMA (33)	−	+/−	+	−	−
CGRP (33)	−	−	−	+	−
C-kit	+	−	−	−	+
CD34	+	−	−	−	+
Tryptase	−	−	−	−	+
Ano1	+	−	−	−	
Connexin 43	+	−	−	−	
Vimentin	+	+	−	−	
Desmin	+	+	−	−	
PDGFRβ	+	+	+		

Ano1 has been shown to be a specific marker for ICC [38], [39]. It has also been reported to be a highly sensitive and specific marker for GIST, with 100% sensitivity compared with 95% for kit signaling [63]. Furthermore we show clear evidence for expression of gap junction protein connexin 43, three mesenchymal related markers in vimentin, desmin and PDGF-R and finally a novel neural marker that has been reported in GIST ICC cells, merlin/NF2. Combining our findings in this study with our previously published data on NTPDase2 positive cells in mouse bladder [33], we summarize the cellular protein expression profile in table 1 and compared these markers with other cell types in the urinary bladder. The picture which emerges strongly indicates that the NTPDase2-positive cells are mouse bladder ICC. This expanded molecular profile may be useful for identifying ICC in mice given the technical difficulties associated with c-kit detection.

Connexin 43 appears to be an important participant in disease states like bladder carcinoma and bladder overactivity, but it is almost universally considered to be located on bladder smooth muscle cells or myofibroblasts [27], [28], [29], [30], [31], [32], [64]. As but one example, connexin 43 was recently shown to be important in regulating diurnal changes in bladder capacity and micturition [65]. While the major findings are not in question and indeed are of great significance, the authors attributed this function to connexin 43 in bladder smooth muscle. The connexin 43 labeling showed a sparse staining pattern that is likely located at the edge of muscle bundle, exactly the location of ICC [33]. Since the presence of ICC in mouse bladder has been difficult to define by c-kit staining, it is understandable that immunostaining deep within the detrusor is usually considered to be smooth muscle cells. However, we have previously shown that these cells are distinct from smooth muscle cells with multiple markers, including α-SMA, Entpd1, Nt5e, NTPDase2 (table 1) [33], and now several more in this study. In other published studies, connexin 43 found in the lamina propria, are attributed as myofibroblasts. In our experience, myofibroblasts are enriched immediately beneath the urothelial layer instead of next to smooth muscle layer and this was demonstrated by labeling myofibroblasts with α-SMA while the connexin 43 positive cells do not stain α-SMA [33]. The finding of connexin 43 in ICC indicates that its function in bladder is likely due to the activity of ICC.

A somewhat surprising finding was that merlin/NF2, most often considered a neuronal marker, was expressed in bladder ICC. Does this indicate that bladder ICC have nerve cell like function? Considering the pacemaker and neurotransducer functions of gastrointestinal ICC, it may be true. The role of ICC in bladder is not well defined but by analogy with GI tract may be responsible for tonic regulation of smooth muscle contractility. Since dysregulation of ICC in GI tract leads to serious pathology ranging from GIST to motility disorders, there is a strong likelihood of ICC involvement in certain syndromes of voiding dysfunction. Indeed as noted, alterations to connexin 43 expression have been linked to cancer and overactive bladder. Better ways to define the morphological signature of ICC will hopefully assist investigations into their function.

In summary, we defined a unique set of molecular markers of NTPDase2 positive cells in mouse urinary bladder. These findings are in line with other reports in gastrointestinal ICC or previous observations in bladder ICC. We believe that, as in other species, these NTPDase2 positive cells are mouse ICC, and we hope that our findings will help other investigators to avoid misidentifying this unique cell type. A clear definition of molecular expression in bladder ICC will also facilitate directed investigations in mouse knockouts to understand the role of ICC in spontaneous contraction and mechanosensation.
